# Complete Genome Sequences of 11 Staphylococcus pseudintermedius Isolates from Dogs in the United States

**DOI:** 10.1128/mra.00002-23

**Published:** 2023-03-06

**Authors:** Ashkan Roozitalab, Ola Elsakhawy, Lufuno Phophi, Stephen A. Kania, Mohamed A. Abouelkhair

**Affiliations:** a Department of Biomedical and Diagnostic Sciences, University of Tennessee, Knoxville, Tennessee, USA; University of Rochester School of Medicine and Dentistry

## Abstract

We report here the genome sequences of 11 canine Staphylococcus pseudintermedius isolates from New York, New Hampshire, California, Pennsylvania, and Kansas. The sequencing information will enable spatial phylogenetic comparisons of staphylococcal species and other related species and will help in better understanding their virulence potential.

## ANNOUNCEMENT

Staphylococcus pseudintermedius is a Gram-positive, coagulase-positive bacterium that can secrete immune-modulating virulence and adhesion factors that facilitate its growth and survival (1). It is an opportunistic canine pathogen, colonizes up to 90% of healthy dogs, and has zoonotic potential. In dogs, it is primarily an etiological agent of skin and wound infections but is also associated with canine otitis externa, pyoderma, and infectious respiratory disease complex (2, 3). Human nasal mucosa may be transiently colonized by S. pseudintermedius (4). It is an occasional opportunistic pathogen in humans, most frequently associated with bite wounds, but it may also cause generalized infections, especially in immunocompromised people (5).

The use of antimicrobials in dogs is associated with emerging antimicrobial resistance (3). S. pseudintermedius is a link in the chain of emerging drug resistance because it is frequently multidrug resistant, possesses virulence factors, and is transmissible to humans. The variability of genotypic features, such as phenotypic characteristics, virulence, and molecular epidemiology, has not been studied in depth in this organism (6–9). Accurate pathogen identification is essential for the clinical treatment of infections (10). The whole-genome sequence availability of different isolates enables genetic confirmation of phenotypic tests and the discovery of new genetic targets for interspecies comparisons.

S. pseudintermedius isolates were obtained from private, state, and university-associated veterinary diagnostic laboratory collections in the United States from samples submitted from dogs for diagnostic purposes. They were obtained for a multicenter epidemiology study. All samples were deidentified to remove patient and owner information. Samples were stored in cryopreservative at −80°C. Pure isolates of S. pseudintermedius were grown on Columbia blood agar plates with 5% sheep blood (Remel) at 37°C. Matrix-assisted laser desorption ionization–time of flight mass spectrometry (MALDI-TOF MS; Bruker) was used to identify the isolates. S. pseudintermedius species assignment was made when log_score_ values were >2. For whole-genome sequencing, bacterial isolates were cultured in tryptic soy broth (TSB) at 37°C overnight, and genomic DNA was extracted using DNeasy UltraClean microbial kits (Qiagen, Germany) according to the manufacturer’s instructions. The quality and quantity of DNA were evaluated using a NanoDrop 2000 spectrophotometer (Thermo Fisher Scientific, USA) and Qubit fluorometer (Fisher, Waltham, MA), respectively. Following the manufacturer’s protocols, for short reads, a genomic library was constructed using a Nextera XT library prep kit and sequenced using a MiniSeq reagent kit in 150-bp paired-end mode on a MiniSeq instrument (Illumina, San Diego, CA). FastQC v0.11.9 was used for read quality control, and the BBDuk v38.84 trimmer was used for trimming and filtering the raw reads. The *de novo* assembly algorithm of CLC Genomics Workbench v22 (CLC bio, Qiagen) was used to *de novo* assemble the trimmed Illumina reads. The NCBI Prokaryotic Genome Annotation Pipeline v6.1 was used to annotate each strain. Multilocus sequence types (MLSTs) were assigned using PubMLST (https://pubmlst.org/; accessed June 2022) (11). Default parameters were used for all software.

The genomic features are presented in Table 1. The average read *N*_50_ value was 88,744 bp. The median values were 2.659 Mbp for the genome size (range, 2.45 to 2.72 Mbp) and 37.3% GC content.

**TABLE 1:** Characteristics and accession numbers for high-quality genome assemblies for 11 Staphylococcus pseudintermedius isolates from the United States

ID^*a*^	MLST^*b*^	Source	Yr of isolation	State of isolation	GenBank accession no.	No. of contigs	Contig *N*_50_ (bp)	Genome size of all contigs (bp)	GC content (%)	SRA accession no.	No. of reads	Cov^*c*^ (×)
CU6	ST1226	Canine ear	2019	NY	JANPRJ000000000	38	266,512	2,544,199	37.5	SRR20462125	2,232,119	10
CU21	ST181	Canine deep skin infection	2019	NY	JANPRI000000000	118	58,386	2,675,297	37.3	SRR20462124	708,098	10
NH3	UNK^*d*^	Canine ear	2020	NH	JANPRR000000000	105	69,430	2,456,686	37.7	SRR20462136	489,752	10
NH4	UNK	Canine skin	2020	NH	JANPRQ000000000	67	113,714	2,499,694	37.6	SRR20462135	1,359,801	10
NH5	ST825	Canine skin/wound	2020	NH	JANPRP000000000	243	26,813	2,725,158	37.2	SRR20462131	452,520	10
PSU13	ST64	Canine skin	2019	PA	JANPRH000000000	134	66,249	2,707,427	37.2	SRR20462134	692,196	10
KSU4	UNK	Canine urine	2019	MO	JANPRG000000000	80	75,441	2,513,611	37.6	SRR20462133	689,700	10
KSU20	ST551	Canine skin/wound	2019	KS	JANPRF000000000	96	89,735	2,659,155	37.3	SRR20462132	2,253,424	10
UCD2	ST181	Canine skin	2019	CA	JANPRM000000000	66	97,955	2,693,573	37.2	SRR20462128	1,334,812	10
UCD6	ST181	Canine ear	2019	CA	JANPRL000000000	198	27,983	2,708,487	37.2	SRR20462127	511,985	10
UCD10	UNK	Canine skin	2019	CA	JANPRK000000000	79	83,973	2,539,162	37.5	SRR20462126	647,500	10

a ID, identifier.

b MLST, multilocus sequence type; ST, sequence type.

c Cov, coverage.

d UNK, unknown.

### Data availability.

The genome sequence assemblies of the isolates have been deposited at DDBJ/ENA/GenBank under the accession numbers listed in Table 1.
